# A New Metric Quantifying Chemical and Biological Property of Small Molecule Metabolites and Drugs

**DOI:** 10.3389/fmolb.2020.594800

**Published:** 2020-12-15

**Authors:** Chuanbo Huang, Yuan Zhou, Jichun Yang, Qinghua Cui, Yanhui Li

**Affiliations:** MOE Key Laboratory of Cardiovascular Sciences, Department of Biomedical Informatics, Department of Physiology and Pathophysiology, Center for Noncoding RNA Medicine, School of Basic Medical Sciences, Peking University, Beijing, China

**Keywords:** drug, chemical small molecule, metabolites, druggable property, normalized bond energy

## Abstract

One prominent class of drugs is chemical small molecules (CSMs), but the majority of CSMs are of very low druggable potential. Therefore, it is quite important to predict drug-related properties (druggable properties) for candidate CSMs. Currently, a number of druggable properties (e.g., logP and pKa) can be calculated by *in silico* methods; still the identification of druggable CSMs is a high-risk task, and new quantitative metrics for the druggable potential of CSMs are increasingly needed. Here, we present normalized bond energy (NBE), a new metric for the above purpose. By applying NBE to the DrugBank CSMs whose properties are largely known, we revealed that NBE is able to describe a number of critical druggable properties including logP, pKa, membrane permeability, blood–brain barrier penetration, and human intestinal absorption. Moreover, given that the human endogenous metabolites can serve as important resources for drug discovery, we applied NBE to the metabolites in the Human Metabolome Database. As a result, NBE showed a significant difference in metabolites from various body fluids and was correlated with some important properties, including melting point and water solubility.

## Introduction

Research and development of pharmaceuticals is a resource-consuming and long process with a variety of challenging risks (Szewczak et al., 2020). Chemical small molecules (CSMs) represent a big class of drugs which mainly function by binding with disease-related target molecules (Wishart et al., 2018a). Given the huge space of target molecules and CSMs, evaluating the druggable potential of both targets (Jung and Kwon, 2015; Liu et al., 2016; Floris et al., 2018; Sztuba-Solinska et al., 2019) and CSMs (Sun et al., 2016; Ashenden et al., 2017; Chitre et al., 2019; Heitmeier et al., 2019; Bhattacharjee et al., 2020) is thus one of the key points of drug discovery. For CSMs, it is known that a number of drug-related properties (druggable properties) affect their druggable potential, for example, human intestinal absorption (HIA), blood–brain barrier (BBB) penetration (Blake, 2000), and some pharmacokinetic properties (Ferreira and Andricopulo, 2019). Therefore, it is crucial to accurately predict druggable properties for an early-phase candidate CSM and large-scale druggable CSM screening.

For the above purpose, a number of *in silico* methods or metrics have already been proposed. For example, properties of logP, logD, logS, logW, and pKa can be calculated using the free online tool ALOGPS (Tetko and Tanchuk, 2002). ChemAxon, a tool that provides solutions and services for chemistry and biology (ChemAxon, 2020), can be used to predict druggable properties such as water solubility, polar surface area (PSA), H bond acceptor count, H bond donor count, and pKa. In addition, given that CSM transport is a key attribute toward better drug potential, several metrics have been presented to evaluate CSM transport properties, including octanol/water partition coefficient, molecular size and shape, hydrogen-bonding capabilities, and topological PSA (van De Waterbeemd et al., 1996; van de Waterbeemd, 1998; Winiwarter et al., 1998; Ertl et al., 2000). These *in silico* methods or metrics provide support for quickly quantifying CSM properties in order to evaluate their druggable potential. However, due to the huge complexity of both biology and chemistry, these methods or metrics are still far from solving all problems in drug research and development. For example, when chemical structures are diverse and complex, the molecular-transport-related physicochemical metric descriptors introduced above may not be reliable enough to predict molecular transport properties (Artursson et al., 1996). Thus, it is necessary to present new *in silico* methods or metrics to quantify druggable properties of CSMs.

We previously revealed that the free energy of the RNA secondary structure has a significant contribution to the importance score of both protein-coding RNA molecules (mRNAs) and noncoding RNAs (lncRNAs and miRNAs) (Zeng et al., 2018; Song et al., 2019). Based on the above observations, we hypothesized that the energy status of CSMs could also represent some properties of these molecules. To confirm this hypothesis, here, we present normalized bond energy (NBE), a new metric. Moreover, we reveal here that the NBE score can significantly represent some critical druggable properties—such as logP, pKa, permeability, BBB penetration, and HIA. Additionally, given that the human endogenous metabolites could be explored as a resource for drug discovery (Bofill et al., 2019), we calculated the NBE scores for CSMs in the human metabolome and performed a comprehensive bioinformatic analysis for the relations between NBE score and other properties of these endogenous metabolic small molecules.

## Materials and Methods

### Datasets of CSMs

We obtained the structural data in SDF format for CSMs from the DrugBank database (Wishart et al., 2018a) (Version 5.0), which include approved small molecule drugs and experimental drugs. Biological macromolecular drugs were excluded from the dataset. We obtained the structural data in SDF format for small molecule metabolites from the Human Metabolome Database (HMDB) (Wishart et al., 2018b) as well. Experiment-derived property (e.g., melting point, logP, and pKa) data of CSMs were also curated from the DrugBank and HMDB. For the property of water solubility, CSMs with terms like “insoluble,” “almost insoluble,” “low soluble,” “mostly insoluble,” “non-soluble,” “not soluble,” and “poorly soluble” were assigned as the insoluble group, whereas CSMs with terms like “soluble,” “easily soluble,” “completely soluble,” “freely soluble,” “highly soluble,” and “very soluble” were assigned as the soluble group. In addition, we obtained the Caco-2 monolayer permeability data of 690 CSMs from the study done by van De Waterbeemd et al. (van De Waterbeemd et al., 1996; Palm et al., 1998; Pham The et al., 2011), the BBB penetration data of 1,638 CSMs from the study reported by Kelder et al. (Kelder et al., 1999; Shen et al., 2010), and the HIA data of 598 CSMs from the study done by Shen et al. (Palm et al., 1997; Shen et al., 2010). The structure files of these CSMs in SMILES format were obtained as well.

### Calculation of NBE

**Figure 1:**
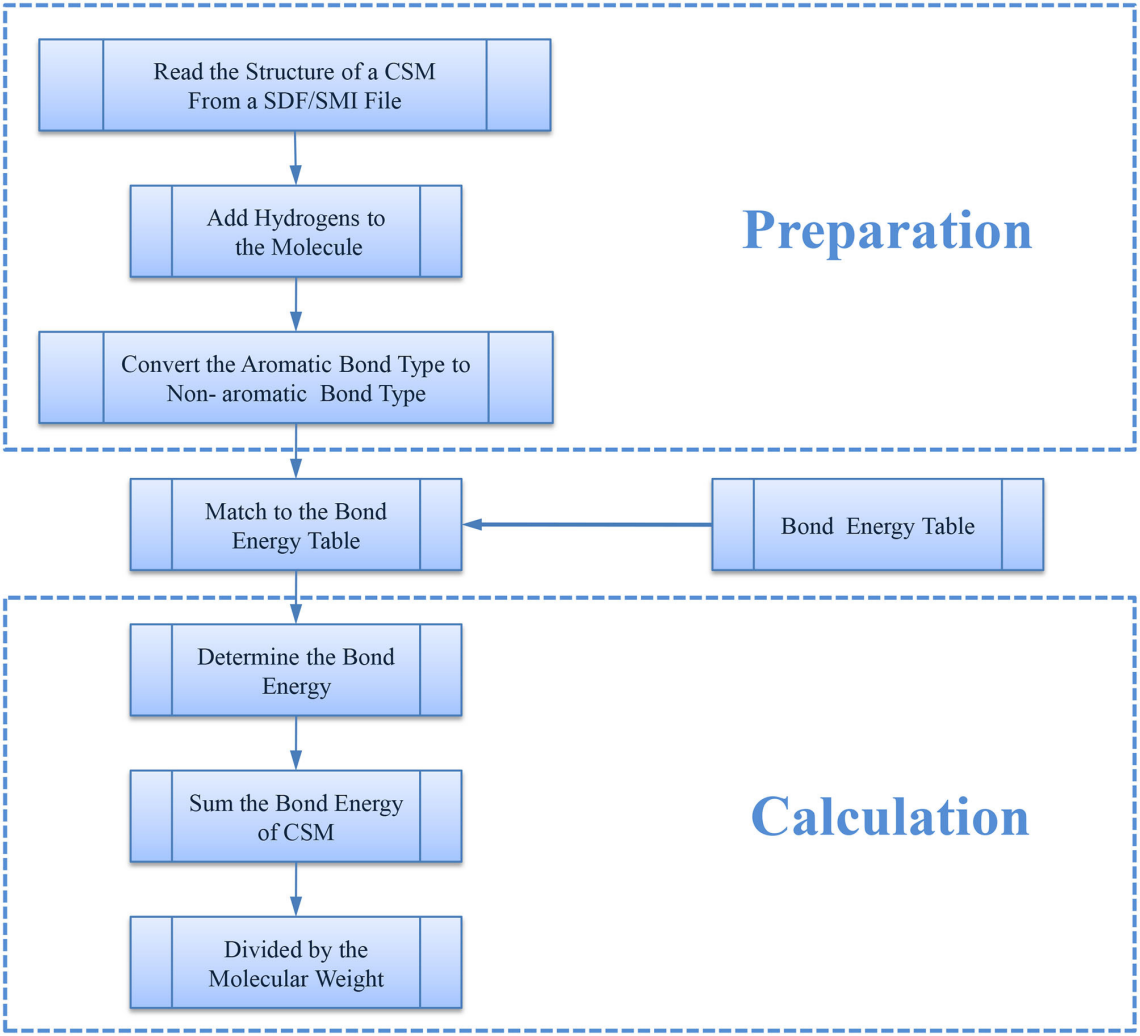
The flowchart of NBE algorithms.

For a representative CSM, we first extracted its bonds from its molecular structure using the RDKit library (Floris et al., 2018) (Version 2017.09.1, 2017) and then determined the energy (kJ/mol) of each bond by matching it with the bond energy table (Supplementary Table 1) through the bond type and the atom type. The bond type here includes single bond, double bond, and triple bond, which denotes the number of shared electron pairs between the two corresponding atoms. Bond energy is defined as a parameter that physically quantifies the strength of a chemical bond and can be measured by the amount of energy required to break a bond. In general, bond energy is the average value of bond dissociation energy of a mole of molecule in the gas phase, typically at a temperature of 298 K. Given that the bigger molecules usually have more bonds and thus would have larger bond energy, we next defined NBE. Here, the original bond energy is normalized using molecular weight (MW), which was calculated using RDKit. The algorithm for the procedure is shown in Figure 1.

Then, NBE can be calculated using the following equation.

(1) $$\begin{array}{r} NBE=\frac{\sum_{i=1}^{n}Bond\;Energy\left(i\right)}{MW} \end{array}$$

where *Bond Energy* (*i*) is the bond energy of bond *i, n* is the number of bonds, and *MW* is the molecular weight of the CSM.

### Statistical Computation

We implemented the algorithm of NBE (http://www.cuilab.cn/nbe/nbe.zip) using Python. Spearman's correlation analysis, *t*-test, and Wilcoxon test were performed using R studio.

## Results

### Global Distribution of NBE Scores

**Figure 2:**
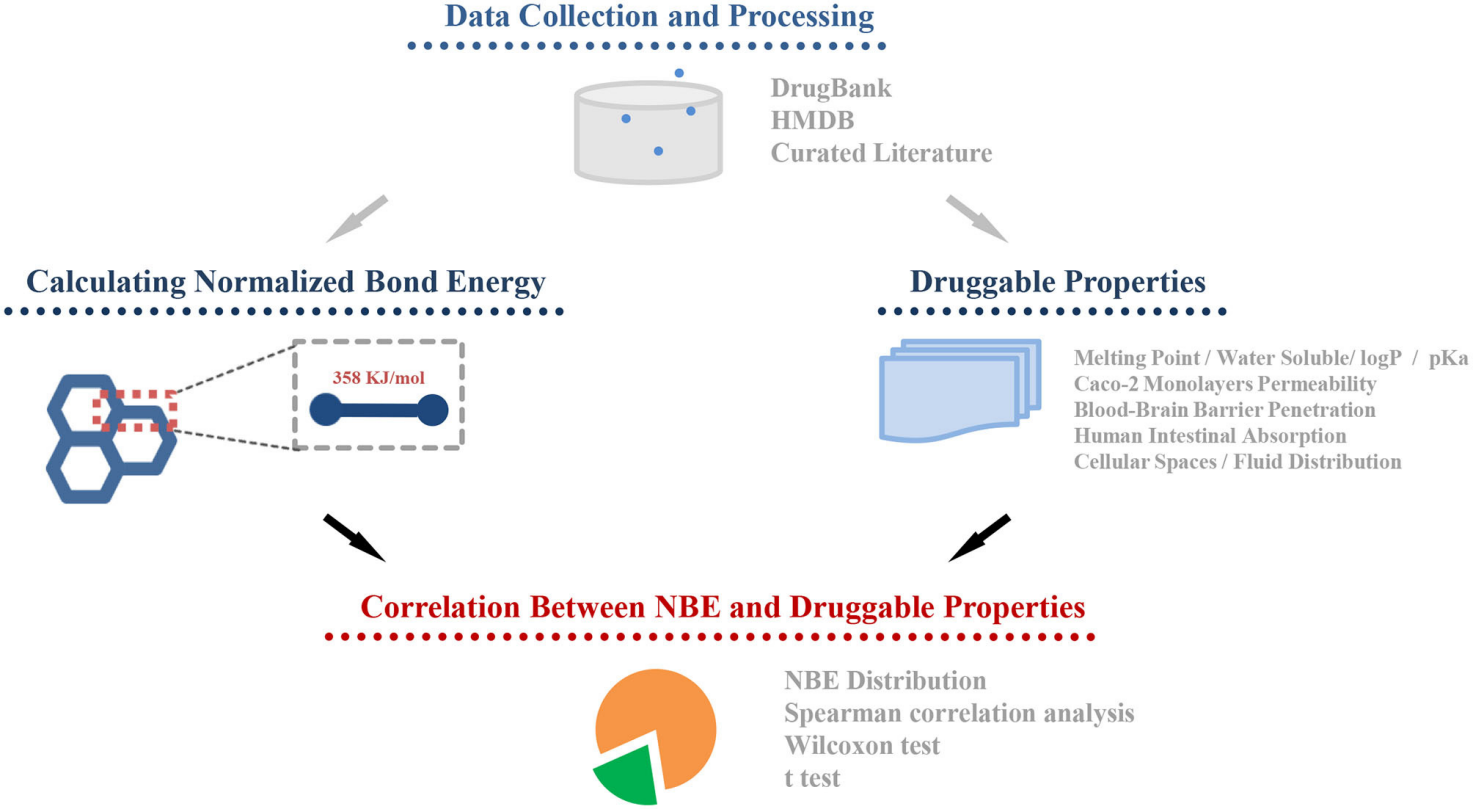
The framework of the whole study.

**Figure 3:**
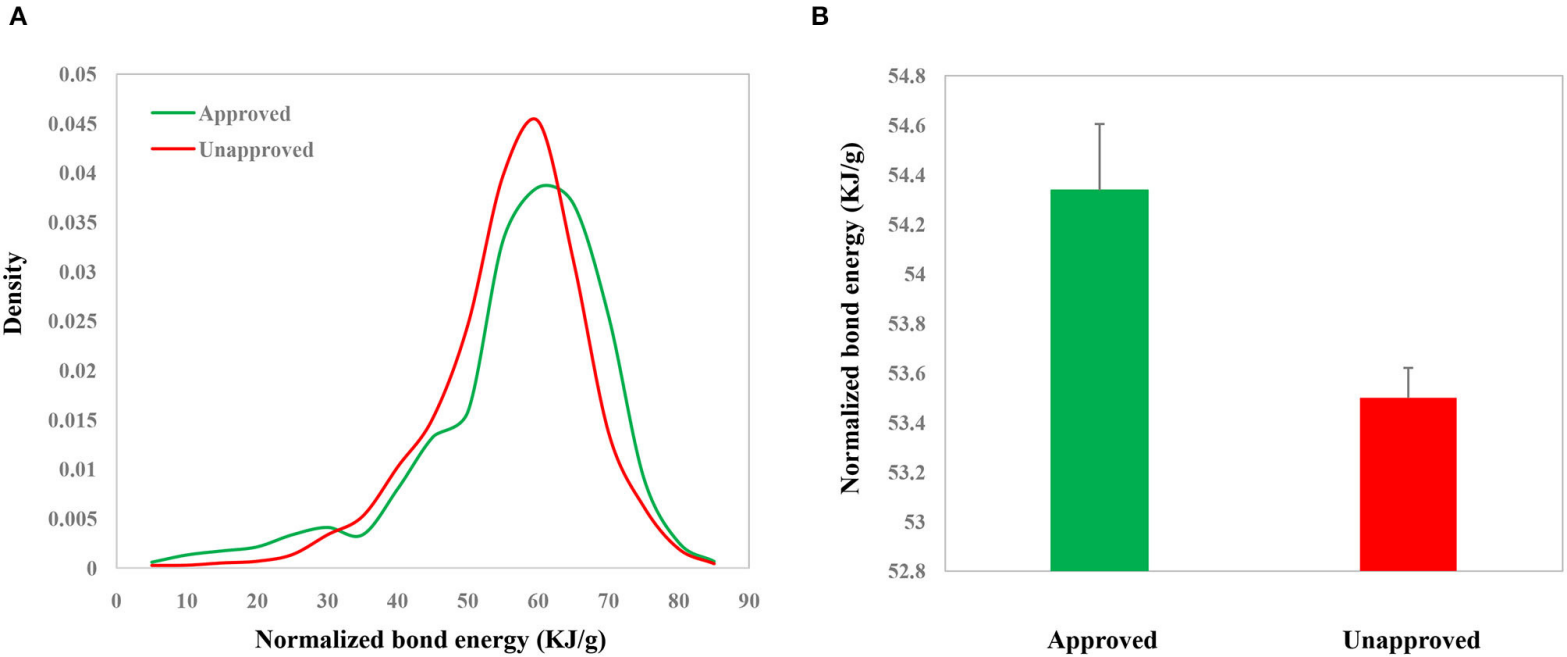
Distributions **(A)** and values **(B)** of the NBE scores of approved chemical small molecules (CSMs) and unapproved ones in DrugBank.

The whole framework of this study is shown in Figure 2. We calculated NBE scores for 10,426 CSMs (2,444 are approved drugs and 7,982 are experimental drugs) from DrugBank and 113,878 human metabolic CSMs from HMDB. The distributions of the DrugBank NBE scores are shown in Figure 3A. The approved drugs have greater NBE scores than the unapproved drugs (*p*-value = 1.17e−13, Wilcoxon test; Figure 3B).

### Correlations of NBE Scores With Experimentally Identified Properties of CSMs in DrugBank

**Figure 4:**
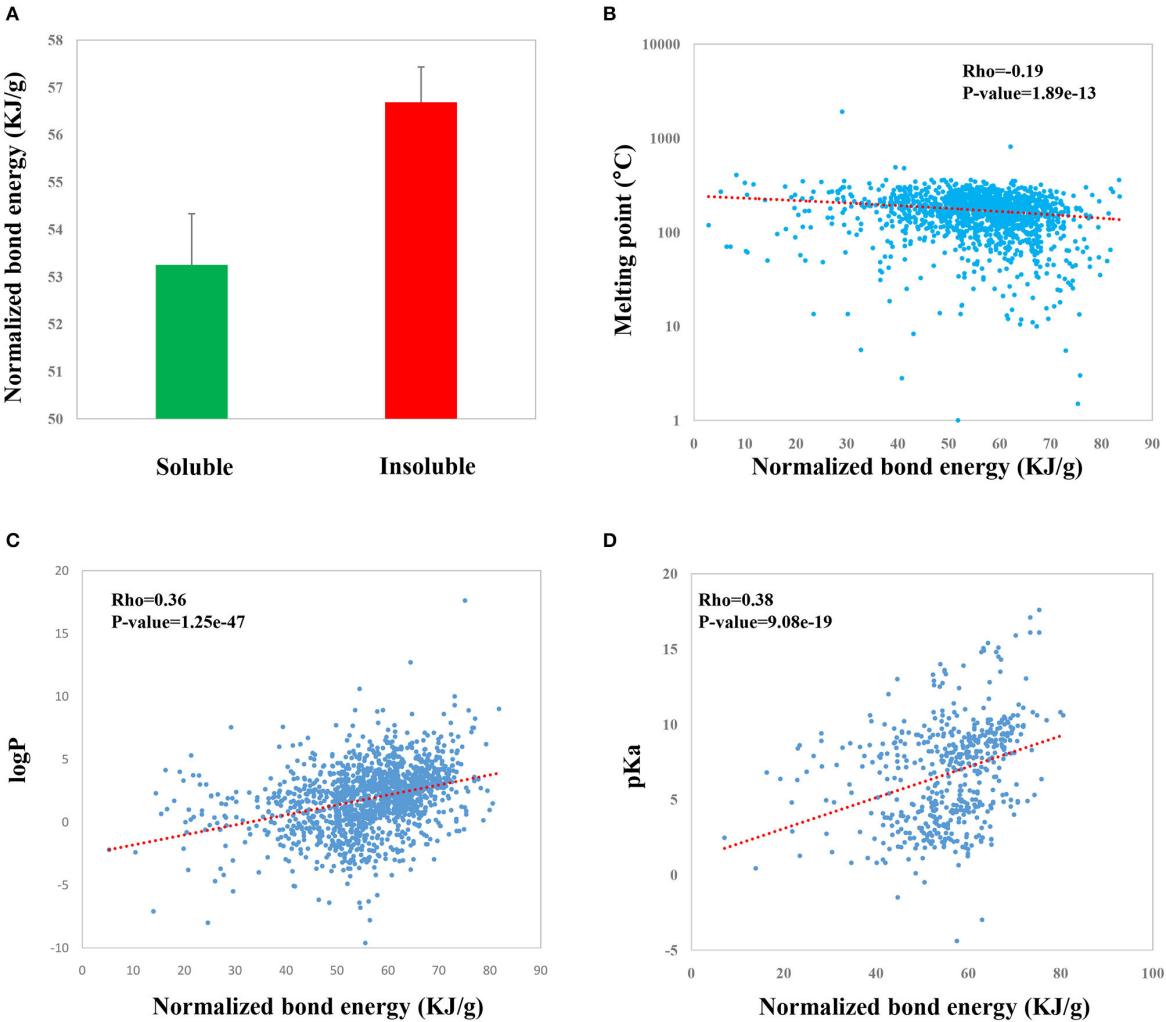
Correlations of the NBE scores of CSMs in DrugBank with druggable properties including water solubility **(A)**, melting point **(B)**, logP **(C)**, and pKa **(D)**.

As a result, we found that NBE score is significantly associated with a number of experimentally identified properties of CSMs in DrugBank. First, we evaluated if there is a difference in NBE scores of the soluble CSMs and the insoluble ones. The results showed that the soluble CSMs have smaller NBE scores compared to the insoluble ones (mean: 53.3 vs. 56.7, *p*-value = 0.009, *t*-test; Figure 4A). Further, we investigated the relations of NBE score with the melting point, logP, and pKa. We found that NBE score shows a significantly negative correlation with melting point (Rho = −0.19, *p*-value = 1.89e−13, Figure 4B) but shows a positive correlation with logP (Rho = 0.36, *p*-value = 1.25e−47, Figure 4C) and pKa (Rho = 0.38, *p*-value = 9.08e−19, Figure 4D).

### NBE Is Correlated With Permeability, BBB Penetration, and HIA

**Figure 5:**
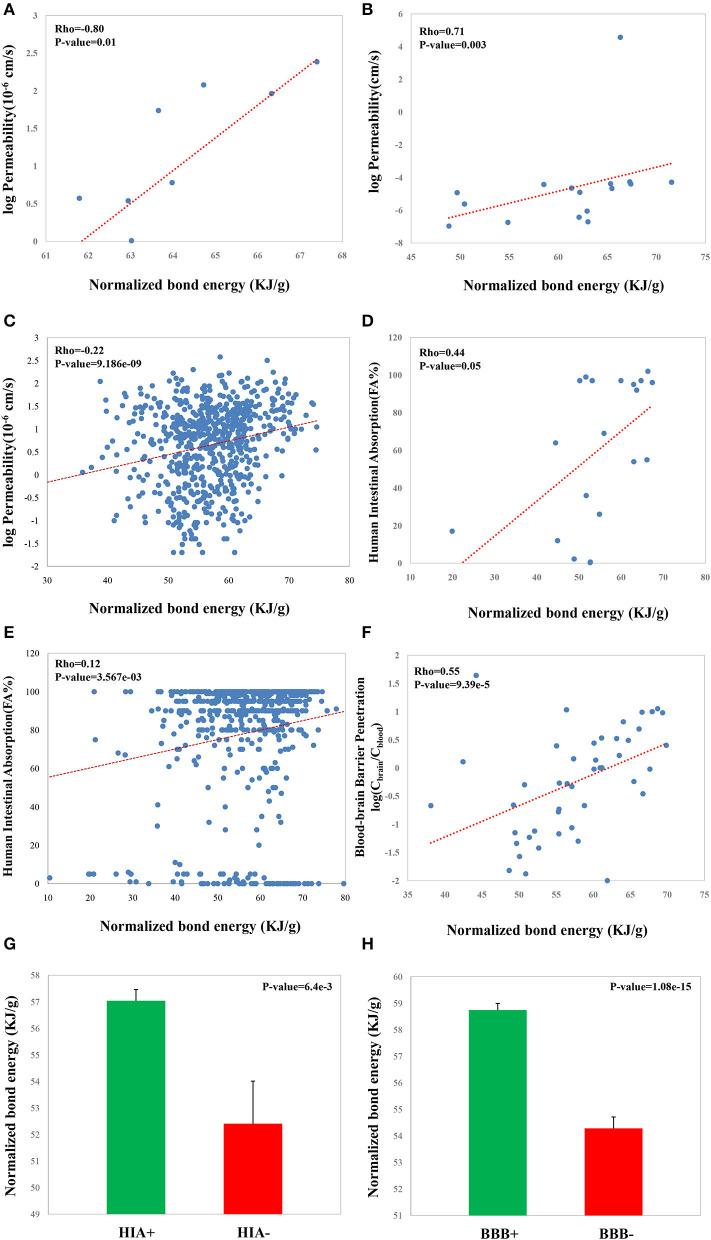
Correlations of the NBE scores of CSMs with some druggable properties, including Caco-2 monolayer permeability from van De Waterbeemd et al. (1996) **(A)**, Palm et al. (1998) **(B)**, and Pham The et al. (2011) **(C)**; HIA from Palm et al. (1997) **(D)** and Shen et al. (2010) **(E)**; and BBB penetration from Kelder et al. (1999) **(F)**. Values of the NBE scores of HIA+ CSMs and HIA– ones from Shen et al. (2010) **(G)**. Values of the NBE scores of BBB+ CSMs and BBB– ones from Kelder et al. (1999) **(H)**.

Permeability, BBB penetration, and HIA are the three critical properties which greatly affect the transport properties of a CSM. We observed significant correlations between NBE and Caco-2 monolayer permeability (Rho = 0.80, *p*-value = 0.01, Figure 5A; Rho = 0.71, *p*-value = 0.003, Figure 5B; Rho = 0.22, *p*-value = 9.186e−09, Figure 5C), between NBE and HIA (Rho = 0.44, *p*-value = 0.05, Figure 5D, Rho = 0.12, *p*-value = 3.567e−03, Figure 5E), and between NBE and BBB penetration (Rho = 0.55, p-value = 9.39e−05, Figure 5F). Moreover, using the FA% value (the oral drug absorption in humans) threshold of 30%, we divide CSMs of the HIA dataset into absorbable (HIA+) terms and nonabsorbable (HIA–) terms. We observed that HIA+ CSMs have bigger NBE scores than the HIA– CSMs (mean: 57.0 vs. 52.4, *p*-value = 0.0064, *t*-test; Figure 5G). Whether CSMs can penetrate the BBB has been recognized as one of the most critical issues for designing drugs targeting the central nervous system. We also observed that BBB penetrable (BBB+) CSMs have bigger NBE scores than the impenetrable (BBB–) CSMs (mean: 58.7 vs. 54.2, *p*-value = 1.08e−15, *t*-test; Figure 5H).

### NBE Is Correlated With Properties of Human Metabolites

**Figure 6:**
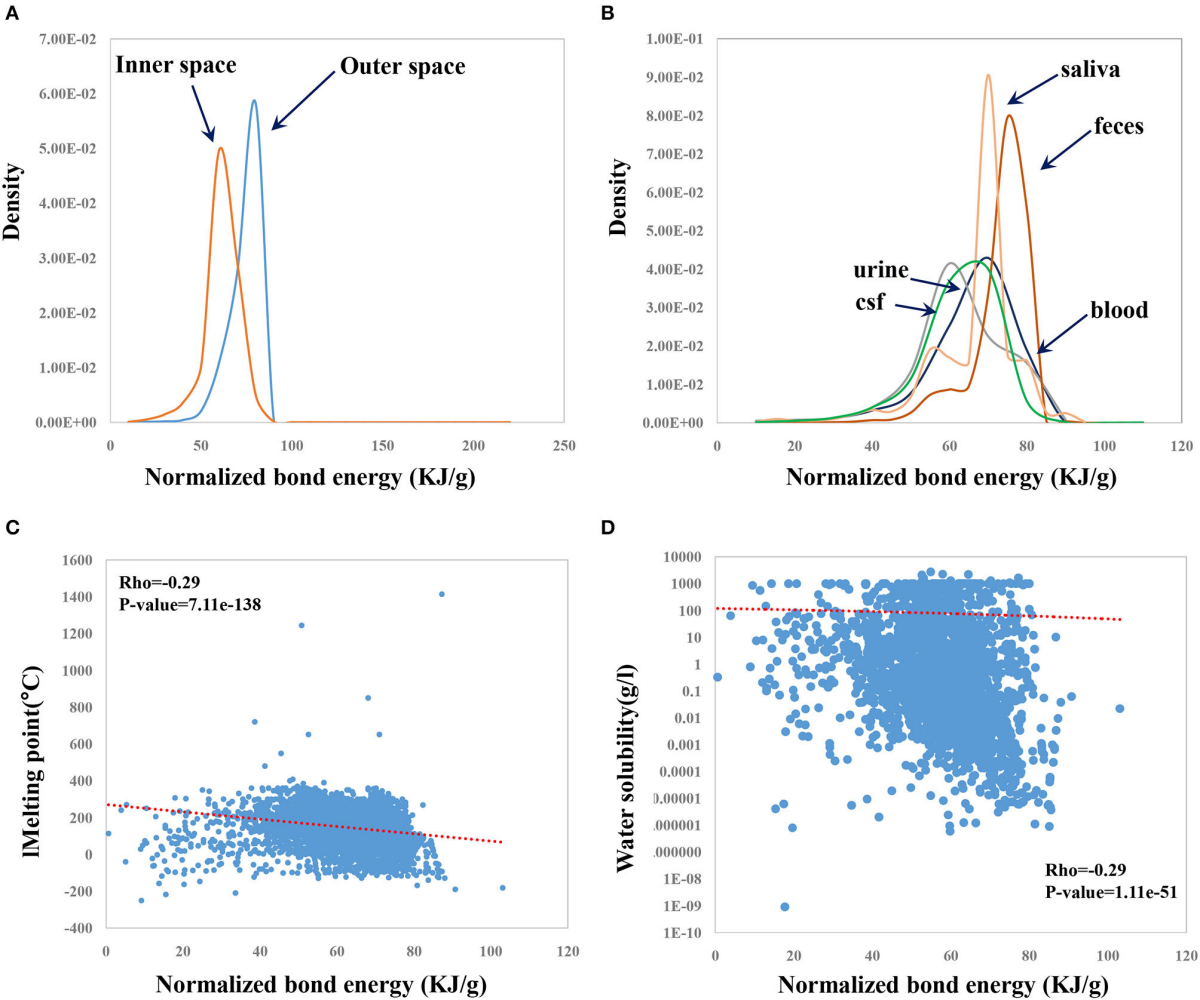
Distributions of the NBE scores of metabolites in different cellular locations **(A)** and metabolites from different body fluids **(B)** and correlation of the NBE scores with melting point **(C)** and with water solubility **(D)** in HMDB.

Natural products represent one important class of CSMs with high druggable potential. The human endogenous metabolites—a type of natural product—could be explored as a resource for drug discovery. It is thus important to investigate whether NBE can describe some properties of human metabolites. We previously revealed biased subcellular distributions for miRNA target genes and sex-biased genes. We found that miRNAs prefer to target genes located within the inner cellular space compared to genes located in the outer cellular space (Cui et al., 2006). Female-biased genes are enriched in the outer cellular space, whereas male-biased genes are enriched in the inner cellular space (Guo et al., 2018). It was considered of much interest to investigate whether there exists a difference in NBE for metabolites in different cellular spaces. The results showed that metabolites in the outer cellular space (metabolites in the extracellular space and/or membrane) have greater NBE scores than those in the inner cellular space (metabolites in the cytoplasm and/or nucleus) (*p*-value = 0, Wilcoxon test; Figure 6A). Moreover, metabolites derived from different body fluids showed a significant difference in NBE scores (*p*-value = 0.0, ANOVA, Figure 6B). Metabolites from feces and saliva showed the highest NBE scores (Figure 6B). In addition, NBE scores of metabolites were correlated with melting point (Rho = −0.29, *p*-value = 7.11e−138; Figure 6C) and water solubility (Rho = −0.29, *p*-value = 1.11e−51; Figure 6D), which is consistent with the results on CSMs in DrugBank.

## Discussion

In this study, we presented a new *in silico* metric, NBE, for quantifying some properties of CSMs. The results showed that NBE is able to describe some critical druggable properties of CSMs. We found that the NBE is correlated with some drug features including logP and pKa, membrane permeability, BBB penetration, and HIA. These metrics are usually used for quantifying the druggable properties of small molecules. For example, logP (octanol–water partition coefficient) is used in drug design as a measure of molecular hydrophobicity, and pKa is related to lipophilicity and the rate/extent of membrane penetration. And other properties including membrane permeability, BBB penetration, and HIA are also utilized for presenting permeability of small molecules.

The BBB separates the brain from the systemic blood circulation and maintains the homeostasis of the central nervous system. Thus, the blood–brain distribution of a CSM is a key characteristic for determining whether it is potentially druggable for the central nervous system or not. HIA is related to the rate of a particular compound crossing the intestinal wall to reach the portal blood circulation. The significant correlated relationships between NBE and BBB penetration and between NBE and HIA provide a simple but efficient metric to quickly judge the potential of a CSM to move into the brain from the circulation and the potential of a CSM to move into the circulation from the intestine.

In addition, we have similar and consistent observations for human metabolites. Interestingly, NBE distributions have a bias for different cellular locations and for different body fluids, which could provide some valuable clues toward metabolite-based drug discovery. However, we found that NBE is not a metric that can be used to judge the druggable potential of candidate small molecule only by a threshold. To use this metric to quantify the druggable potential, researchers of pharmaceuticals need to compare more small molecules with their NBE scores to draw conclusions. In addition, due to the limitation of data sources, the number of CSMs in some datasets used in this study is small and may introduce bias in the accuracy of the results. In summary, this study presented a simple but efficient metric to describe druggable properties of CSMs. The utility of NBE may be improved by combining it with other *in silico* methods or metrics in the future.

## Data Availability Statement

The datasets presented in this study can be found in online repositories. The names of the repository/repositories and accession number(s) can be found in the article/Supplementary Material.

## Author Contributions

JY and QC designed this project. CH implemented the algorithm and wrote the paper. YL and YZ provided data or suggestions in the revision. All authors contributed to the article and approved the submitted version.

## Conflict of Interest

The authors declare that the research was conducted in the absence of any commercial or financial relationships that could be construed as a potential conflict of interest.
